# A User Study on Robot Skill Learning Without a Cost Function: Optimization of Dynamic Movement Primitives via Naive User Feedback

**DOI:** 10.3389/frobt.2018.00077

**Published:** 2018-07-02

**Authors:** Anna-Lisa Vollmer, Nikolas J. Hemion

**Affiliations:** ^1^Applied Informatics Group, Cluster of Excellence Cognitive Interaction Technology (CITEC), Bielefeld University, Bielefeld, Germany; ^2^AI Lab, SoftBank Robotics Europe, Paris, France

**Keywords:** programming by demonstration, imitation learning, CMA-ES, human-robot interaction, DMP, human factors, optimization, skill learning

## Abstract

Enabling users to teach their robots new tasks at home is a major challenge for research in personal robotics. This work presents a user study in which participants were asked to teach the robot Pepper a game of skill. The robot was equipped with a state-of-the-art skill learning method, based on dynamic movement primitives (DMPs). The only feedback participants could give was a discrete rating after each of Pepper's movement executions (“very good,” “good,” “average,” “not so good,” “not good at all”). We compare the learning performance of the robot when applying user-provided feedback with a version of the learning where an objectively determined cost via hand-coded cost function and external tracking system is applied. Our findings suggest that (a) an intuitive graphical user interface for providing discrete feedback can be used for robot learning of complex movement skills when using DMP-based optimization, making the tedious definition of a cost function obsolete; and (b) un-experienced users with no knowledge about the learning algorithm naturally tend to apply a working rating strategy, leading to similar learning performance as when using the objectively determined cost. We discuss insights about difficulties when learning from user provided feedback, and make suggestions how learning continuous movement skills from non-expert humans could be improved.

## 1. Introduction

Robots are currently making their entrance in our everyday lives. To be able to teach them novel tasks, learning mechanisms need to be intuitively usable by everyone. The approach of *Programming by Demonstration* (Billard et al., 2008) includes users to show their robot how a task is done (for example via kinesthetic teaching), and the robot will then reproduce the demonstrated movement. However, not all tasks can be easily demonstrated to a robot this way. For example some tasks are only solved with very precise movements which are difficult to successfully demonstrate for the user. Instead, it is often more feasible to let the robot self-improve from an imperfect demonstration. Most research on robot learning aims primarily at optimizing the final task performance of the robot, while disregarding the usability of the system by non-expert users. In particular, Programming by Demonstration studies and, even more so the optimization, are primarily tested in laboratory environments and rarely evaluated with human users, let alone with non-experts. The typical workflow for creating an optimization system encompasses the definition of a suitable cost function, which the system can evaluate to improve its performance. Finding a cost function that will ensure the desired outcome of the robot learning is far from trivial. In fact, often it is difficult even for domain experts to define a cost function that does not lead to unexpected behaviors by the robot. To be usable by non-expert users, it is unrealistic to expect the user to design a cost function in order to teach their robot a new skill. To make things worse, many cost functions require an external sensory setup (in addition to the robot's on-board sensors) to measure relevant features precisely enough for the computation of the cost function—again, something which is feasible in a laboratory environment, but not realistic for use at home by non-experts.

The general research topic of this work is thus to investigate, whether it is possible to employ a state-of-the-art optimization system in a user-centered setup: one that is intuitively usable by non-experts, and could easily be operated outside the laboratory (for example, it does not require expensive or difficult to calibrate equipment). In particular, we concentrate on robot learning of complex movement skills with a human teacher. As a method, we chose optimization of Dynamic Movement Primitives (DMPs) (see section 2) as a widely used method from the Programming by Demonstration literature.

It is commonly assumed that the feedback humans provide is a noisy and unreliable reward signal (e.g., Knox and Stone, 2012; Weng et al., 2013; Daniel et al., 2015): it is assumed that humans do not provide an optimal teaching signal, and therefore additional care should be taken when using the human-provided signal in a robot learning system. In contrast, here we deliberately chose to use an unaltered optimization system, without any modifications to the learning algorithm for “dealing with” the human-provided teaching signal or specific adaptations toward the human. In doing so, we aim at demonstrating, as a baseline, the performance of an unaltered, state-of-the-art Programming by Demonstration setup trained using human feedback alone. The only modification in our system is to replace the sensory-based cost evaluation by an intuitive to use graphical user interface, allowing the user to provide a discrete-valued feedback to the robot after each movement execution.

### 1.1. Related work

The field of Interactive Machine Learning (IML) aims to give the human an active role in the machine learning process (Fails and Olsen, 2003). It is a rather vast field including the human in an interactive loop with the machine learner, ranging from web applications to dialog systems, but also robots: the learner shows its output (e.g., performance, predictions) and the human provides input (e.g., feedback, corrections, examples, demonstrations, ratings). In robotics, IML combines research on machine learning (section 1.1.1) and human-robot interaction (section 1.1.2).

#### 1.1.1. Machine learning with human teachers

Regarding machine learning research, there is a large body of literature on incorporating human-provided reward signals into reinforcement learning algorithms. The majority of approaches focuses on the case where the action space of the robot is discrete (e.g., Abbeel and Ng, 2004; Thomaz and Breazeal, 2008; Chernova and Veloso, 2009; Taylor et al., 2011; Cakmak and Lopes, 2012; Griffith et al., 2013; Cederborg et al., 2015), which means that the robot already has to know the “steps” (or “basic actions”) required to solve a task in advance: Related work in this area includes the work of Thomaz et al., who investigated user input to a reinforcement learning agent that learns a sequential task in a virtual environment (Thomaz et al., 2006). They then altered the learning mechanism according to the results of their Human-Robot Interaction (HRI) studies. Also Senft et al. recently presented a study with a virtual reinforcement learning agent learning sequential tasks with user rewards (Senft et al., 2017).

Here, in contrast, we are interested in the case of a continuous action space, which would allow a human user to teach their robot entirely new actions (which could in principle then also be used as new “basic actions” in reinforcement learning methods as the ones just mentioned). There is some existing work on robot learning from user feedback where the robot's action space is continuous. Knox and Stone proposed the “TAMER” framework, aimed at learning a model of the human-provided reward, explicitly taking effects such as time-delayed responses into account (Knox and Stone, 2009). TAMER has mostly been used for learning in the case of discrete state and action spaces (Knox and Stone, 2012; Knox et al., 2012a,b), but recently has also been applied to traditional reinforcement learning benchmark tasks involving continuous spaces (e.g., Vien and Ertel, 2012). Similarly, Daniel et al. use Gaussian process regression and Bayesian optimization in combination with relative entropy policy search to estimate a reward function from user-provided feedback. In contrast to these works, we do not estimate a reward function but directly treat the user responses as teaching signal to the learning algorithm, to evaluate if an unaltered optimization algorithm in conjunction with DMPs can operate on user-provided discrete scores, noisy or not.

Instead of requesting a score or reward value directly from the user, it has been suggested to employ preference-based learning (Christiano et al., 2017; Sadigh et al., 2017): the user is repeatedly presented with two alternative performances by the robot or agent, and is asked to select one over the other. Sadigh et al. used such an approach to let users teach a simulated 2-dimensional autonomous car to drive in a way deemed reasonable by the user (Sadigh et al., 2017). Their system learned a reward function from the human provided reward. However, the function estimation relied on a set of predefined features to succeed in learning from relatively little data. Like designing a cost function, also the design of suitable feature representations for the cost function estimation in itself can be challenging, and certainly is for non-experts. Christiano et al. successively presented pairs of short video clips showing the performance of virtual agents (simulated robots in one task, and agents playing Atari games in another task) to human participants, who then selected the performance that they preferred (Christiano et al., 2017). Using this feedback alone, the virtual agents were able to learn complex behaviors. Christiano et al. also learn a model of the user-provided responses. Interestingly, they were able to reduce the total amount of time humans had to interact with the learning system (watch videos, provide feedback) to only about 1 h. However, their work is based on deep reinforcement learning methodology and thus requires the agent to train in total for hundreds of hours, which poses a severe difficulty for application in real robots on the one hand in terms of time necessary for training, and on the other hand due to other factors such as physical wear down. In contrast, we present a system that does not rely on the definition of suitable feature representations, and can learn successful movement skills from non-expert users in as little as 20 min in total.

#### 1.1.2. Human-robot interaction with machine learners

Developing machine learning algorithms, we cannot imagine or model in theory what everyday, non-expert users will do with the system. For example, studies in imitation learning or Programming by Demonstration have shown that people will show completely different movement trajectories depending on where the robot learner is looking at the time of demonstration Vollmer et al. (2014). Thus, if we develop systems without considering human factors and testing it in HRI studies with everyday people, then our systems in the end might not be usable at all. Here, we briefly review studies of human-robot-learning scenarios with real naive human users. Some related HRI studies test machine learning algorithms with humans users and examine how naive users *naturally* teach robots and how the robot's behavior impacts human teaching strategies (see Vollmer and Schillingmann, 2017, for a review). In the area of concept learning for example, Cakmak and Thomaz (2010) and Khan et al. (2011) studied how humans teach a novel concept to a robot. In a task with simple concept classes where the optimal teaching strategy is known, Cakmak and Thomaz (2010) found that human teachers' strategies did not match the optimal strategy. In a follow-up study, they tried to manipulate the human teacher to employ the optimal teaching strategy. Khan et al. (2011) provided a theoretical account for the most common teaching strategy they observed by analyzing its impact on the machine learner.

Natural human teaching behavior of movement skills is very complex, highly adaptive and multimodal. Previous HRI studies have investigated the naive demonstration of continuous robot movement skills, focusing on the usability of kinesthetic teaching Weiss et al. (2009), or not applying machine learning algorithms but studying the influence of designed robot behavior, for example incorporating findings from adult-infant interactions (Vollmer et al., 2009, 2010, 2014).

Weiss et al. (2009) have shown that naive users are able to teach a robot new skills via kinesthetic teaching. Here, we do not focus on the demonstration part of the skill learning problem, but the users' feedback replaces the cost function for task performance optimization.

### 1.2. Contribution and outline

In this work, we investigate whether a completely unmodified version of a state-of-the-art skill learning algorithm can cope with naive, natural user feedback. We deliberately restricted our system to components of low complexity (one of the most standard movement representations in the robotics literature, a very simple optimization algorithm, a simplistic user interface), in order to create a baseline against which more advanced methods could be compared.

We present a first study with non-expert participants who teach a full-size humanoid robot a complex movement skill. Importantly, the movement involves continuous motor commands and cannot be solved using a discrete set of actions.

We use Dynamic Movement Primitives (DMPs), which are “the most widely used time-dependent policy representation in robotics (Ijspeert et al., 2003; Schaal et al., 2005)” (Deisenroth et al., 2013, p. 9) combined with Covariance Matrix Adaptation Evolution Strategy (CMA-ES, Hansen, 2006) for optimization. Stulp and Sigaud (2013) have shown that the backbone of CMA-ES, “(μ_*W*_, λ)-ES one of the most basic evolution strategies is able to outperform state-of-the-art policy improvement algorithms such as PI^2^ and PoWER with policy representations typically considered in the robotics community.”

The task to be learned is the ball-in-cup game as described by Kober and Peters (2009a). Usually, these state-of-the-art learning mechanisms are tested in the lab in simulation or with carefully designed cost functions and external tracking devices. Imagine robots in private households that should learn novel policies from their owners. In this case, the use of external tracking devices is not feasible, as it comes with many important requirements (e.g., completely stable setup and lighting conditions for color-based tracking with external cameras). We chose the ball-in-cup game for our experiment, because it has been studied in a number of previous works (Miyamoto et al., 1996; Arisumi et al., 2005; Kober and Peters, 2009b; Nemec et al., 2010, 2011; Nemec and Ude, 2011) and we can therefore assume that it is possible to solve the task using DMP-based optimization. Still, it is not at all trivial to achieve a successful optimization, but a carefully set up sensory system is required to track the ball and the cup during the movement, as well as a robustly implemented cost function (covering all contingencies, see section 2.2). We therefore believe the task to be a suitable representative for the study of robot learning of complex movements from naive users, which would otherwise require substantial design effort by an expert.

Policy search algorithms with designed cost functions usually operate on absolute distances obtained via a dedicated sensory system. However, participants in our study are naive in the sense that they are not told a cost function and it is difficult for humans to provide absolute distances (i.e., the cost) as feedback to the robot. Therefore, we provided participants with a simple user interface with which they give discrete feedback for each robot movement on a scale from one to five.

The central question we aim to answer is: can human users without technical expertise and without manual or specific instructions teach a robot equipped with a simple, standard learning algorithm a novel skill in their homes (i.e., without any external sensor system)? For the evaluation, we focus on system performance and the user's teaching behavior. We report important difficulties of making learning in this setup work with an external camera setup (section 2.2) and with human users (section 4.1).

## 2. Materials and methods

### 2.1. System

#### 2.1.1. Robot

Pepper is a 1.2 m tall humanoid robot developed and sold by SoftBank Robotics. Pepper's design is intended to make the interaction with human beings as natural and intuitive as possible. It is equipped with a tablet as input device. Pepper is running NAOqi OS. Pepper is currently welcoming, informing and amusing customers in more than 140 SoftBank Mobile stores in Japan and it is the first humanoid robot that can now be found in Japanese homes.

In our study, Pepper used only its right arm to perform the movements. The left arm and the body were not moving. For the described studies, any collision avoidance of the robot has been disabled. Joint stiffness is set to 70%.

#### 2.1.2. Setup

**Figure 1:**
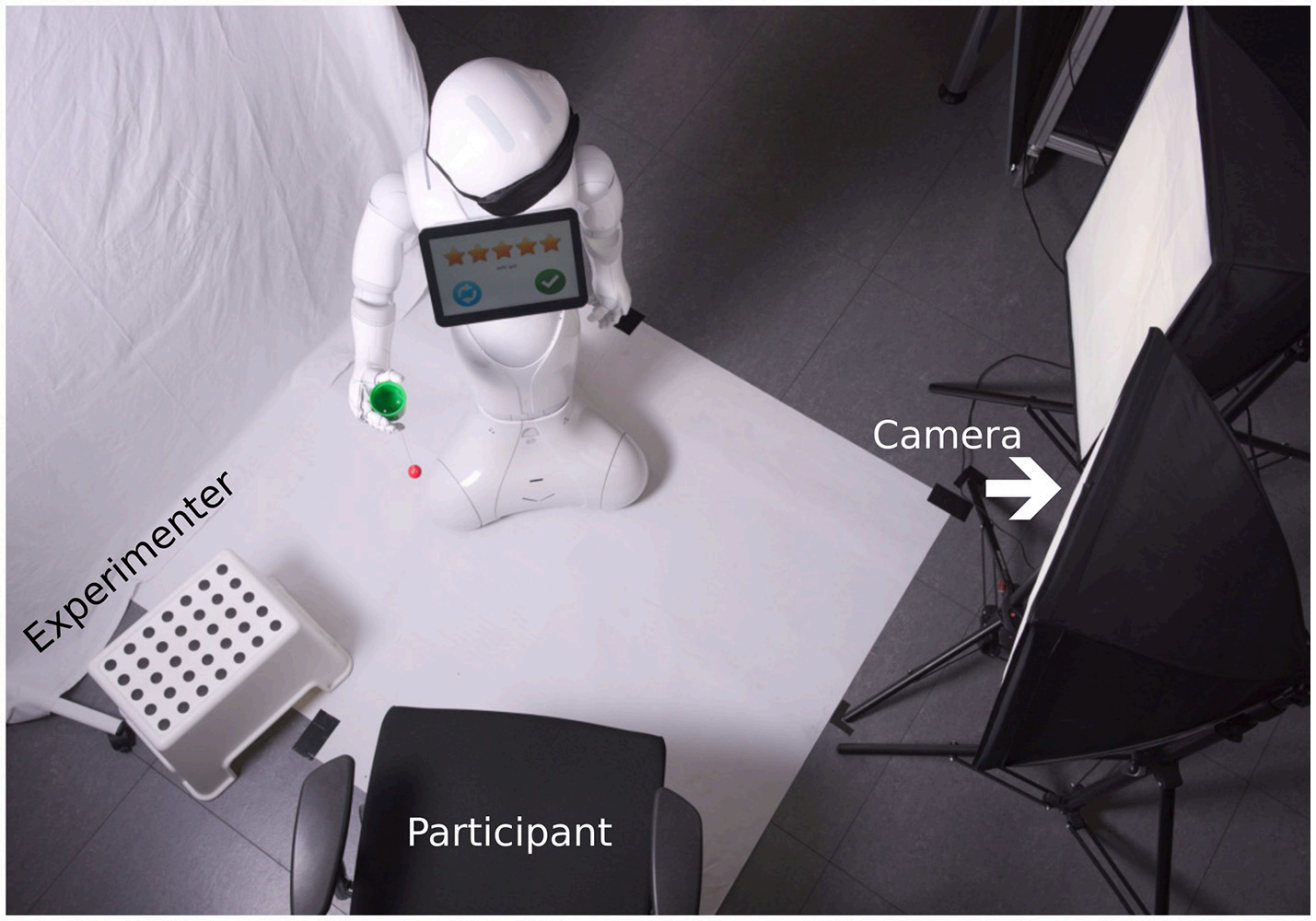
Experimental setup from above. In the studies with optimization via the external camera setup (section 2.2), where the experimenter only returned the ball to its home position, the seat for the participant remained empty.

The setup is shown in Figure 1. Two cameras recorded the movement at 30 Hz, one from above and another one from the side. This allowed for tracking of the ball and cup during the movements. All events, including touch events on the tablet of the robot were logged.

#### 2.1.3. Ball and cup

The bilboquet (or ball and cup) game is a traditional children's toy, consisting of a cup and a ball, which is attached to the cup with a string, and which the player tries to catch with the cup. Kober et al. have demonstrated that the bilboquet movement can be learned by a robot arm using DMP-based optimization (Kober and Peters, 2009a), and we have demonstrated that Pepper is capable of mastering the game^1^. In this study, the bilboquet toy was chosen such that the size of the cup and ball resulted in a level of difficulty suitable for our purposes (in terms of time needed to achieve a successful optimization) and feasibility regarding the trade-off between accuracy (i.e., stiffness value) and mitigating hardware failure (i.e., overheating). Usually, such a movement optimization provides a more positive user experience when learning progress can be recognized. Thus, the initialization and exploration parameters together should yield an optimization from movements somewhere rather far from the cup toward movements near the cup. With a small cup, if the optimization moves rather quickly to positions near the cup, the “fine-tuning” of the movement to robustly land the ball in the cup takes disproportionally long. This is partially due to the variance introduced by hardware. Therefore, we chose the cup size to result in an agreeable user experience by minimizing the time spent on “fine tuning” of the movement near the cup at the end of the optimization process on the one hand, and on the other hand by minimizing the teaching time until the skill has been successfully learned.

#### 2.1.4. Learning algorithm

We implement the robot's movement using dynamic movement primitives (DMPs) (Ijspeert et al., 2013). We define the DMP as coupled dynamical systems:

(1) $$\begin{array}{r} \frac{1}{\tau }{ӱ}_{t}=\alpha_{y}\left(\beta \left(y_{g}-y_{t}\right)-{ẏ}_{t}\right)\;+\;v_{t}\left(y_{g}-y_{0}\right)\cdot h_{\theta }\left(x_{t}\right) \end{array}$$

(2) $$\begin{array}{r} \frac{1}{\tau }{\overset{\cdot }{v}}_{t}=-\alpha_{v}v_{t}\left(1-\frac{v_{t}}{K}\right) \end{array}$$

The “transformation system,” defined in Equation (1), is essentially a simple linear spring-damper system, perturbed by a non-linear forcing term *h*_θ_. Without any perturbation, the transformation system produces a smooth movement from any position *y*_*t*_ toward the goal position *y*_*g*_ (both positions defined in the robot's joint space). The forcing term *h*_θ_ is a function approximator, parametrized by the vector θ. It takes as input a linear system *x*_*t*_, which starts with value 0 and transitions to 1 with constant velocity (see Stulp, 2014). The introduction of the forcing term allows us to model any arbitrarily shaped movement with a DMP.

As suggested by Kulvicius et al. (2012), a “gating system” (defined in Equation 2) is used to ensure that the contribution of the forcing term *h*_θ_ to the movement disappears after convergence. It is modeled after a sigmoid function, with starting state 1 and attractor state 0, where the slope and inflection point of the sigmoid function are determined by the parameters α_*v*_ and *K* (for details, see Stulp, 2014). This way, stable convergence of the system can be guaranteed even for strong perturbations, as we know that the transformation system without any perturbation by the forcing term is stable, and the multiplication of the forcing term with the gating variable *v*_*t*_ blends out the perturbation once the gating system has converged.

For learning the ball-in-a-cup skill on Pepper, we adopt Stulp and Sigaud's method of optimizing the parameter vector θ using simple black-box optimization (Stulp, 2014). More specifically, we use the Covariance Matrix Adaptation Evolution Strategy (CMA-ES, Hansen, 2006) for optimization, and locally weighted regression (Atkeson et al., 1997) for the function approximator *h*_θ_. The parameter space is 150 dimensional as we use 5 degrees-of-freedom (DoF) in the robot arm and 30 local models per DoF. Following the Programming by Demonstration paradigm, we initialize the local models via kinesthetic teaching, thus first recording a trajectory, and subsequently determining model parameters via regression on the trajectory data points. After this initialization, we keep all but one parameter of each local model fixed: in the CMA-ES-based optimization, we only optimize the offset of the local models, which proves to allow for a change in the shape of the trajectory that is sufficient for learning.

CMA-ES functions similarly to a gradient descent. After the cost has been obtained via the defined objective function for each roll-out in a batch, in each update step, a new mean value for the distribution is computed by ranking the samples according to their cost and using reward-weighted averaging. New roll-outs are sampled according to a multivariate normal distribution in ℝ^*n*^ with here, *n* = 150. There are several open parameters which we manually optimized. We aimed at allowing a convergence to a successful movement within a reasonable amount of time. The parameters include the initial trajectory given to the system as a starting point, the number of basis functions the DMP uses to represent the movement, the initial covariance for exploration and the decay factor by which the covariance is multiplied after each update, the batch size as the number of samples (i.e., roll-outs) before each update, the stiffness of the joints of the robot, the number of batches (i.e., updates) for one session in the described studies. The initial trajectory was recorded via kinesthetic teaching to the robot. We chose a trajectory with too much momentum, such that the ball traveled over the cup. All parameters and their values are listed in Table 1.

**Table 1:** Overview of the open parameters of the system which influence learning.

**Parameter**	**Value**
Initialization	Same for all studies
Number of basis functions	30
Covariance	80
Decay rate	0.8
Batch size	10
Stiffness	70 %
Number of batches	8

### 2.2. Optimization—external camera setup

**Figure 2:**
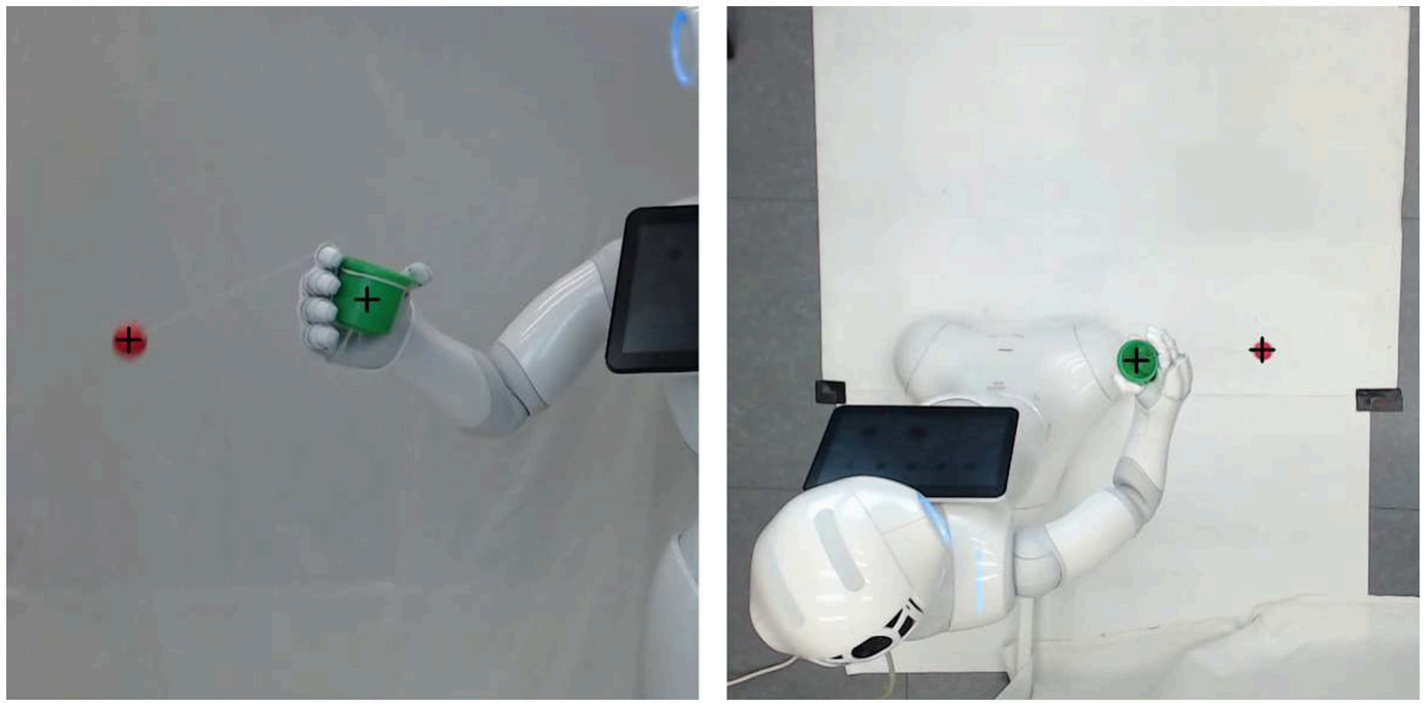
Detection of ball and cup at the respective frame of interest in side and top view.

In order to optimize the movement with external cameras and to create a base-line corresponding to a state-of-the-art skill learning system, a carefully designed cost function is defined that determines the cost as the distance between the ball and the cup at height of the cup when the ball is traveling downward, similar as described in Kober and Peters (2009a). As with any sensory system designed for an automated measurement of a cost or error, significant care has to be taken to ensure robust and accurate performance, as already a slightly unreliable sensory system can prohibit the skill learning. In this case, particular care had to be taken for example in choosing camera models with high-enough frame rates, to ensure that the fast traveling ball could be accurately tracked in the camera image. During a roll-out, the ball typically (this depends on the chosen initialization, here, it will) passes the height of the cup and then descends again. From a webcam recording the side of the movement, we determine the exact frame when the descending ball passes the vertical position of the cup. In the corresponding frame from the top view camera at this moment, we measure the distance between the center of the ball and the center of the cup in pixels (see Figure 2).

We showed a cyan screen on the robot's tablet right before the movement began which could be detected automatically in the videos of both the side and top camera, to segment the video streams. The experimenter repositioned the ball in the home position after each roll-out.

Apart from the usual issues for color-based tracking, as for instance overall lighting conditions, the above heuristic for cost determination needed several additional rules to cover exceptions (for instance, dealing with the ball being occluded in the side view when it lands in the cup or passes behind the robot's arm). More severely, in this particular task the ball occasionally hits the rim of the cup and bounces off. The camera setup in this case detects the frame in which the ball passes beside the cup *after* having bounced off the rim, and thus assigns a too high cost to the movement. Although we were aware of this, we refrained from taking further measures to also cover this particularity of the task, as we found that the camera-based optimization would still succeed. In a version of the game with a smaller cup size however, this proves to be more problematic for the optimization and needs to be taken into account.

For initial trajectories that do not reach the height of the cup, additional rules would need to be implemented for low momentum roll-outs.

### 2.3. Optimization—naive users

In the following, we describe the conducted HRI study with non-expert users, who are naive to the learning algorithm and have little to no experience with robots. It was approved by the local ethics committee and informed consent was obtained from all participants prior to the experiment.

#### 2.3.1. Participants

Participants were recruited through flyers/adds around the campus of Bielefeld University, at children's daycare centers, and gyms. Twenty-six persons took part in the experiment. Participants were age- and gender-balanced (14 f, 12 m, age: *M* = 39.32, *SD* = 15.14 with a range from 19 to 70 years).

#### 2.3.2. Experimental setup

The experiment took place in a laboratory at Bielefeld University. The participant was sitting in front of Pepper. The experimenter sat to the left of the participant (see Figure 1). As in the other condition, two cameras recorded the movement, one from above and another one from the side, such that a ground truth cost could be determined. However, the camera input was neither used for learning, nor was communicated to participants that and how the cost would be determined from the camera images.

#### 2.3.3. Course of the experiment

**Figure 3:**
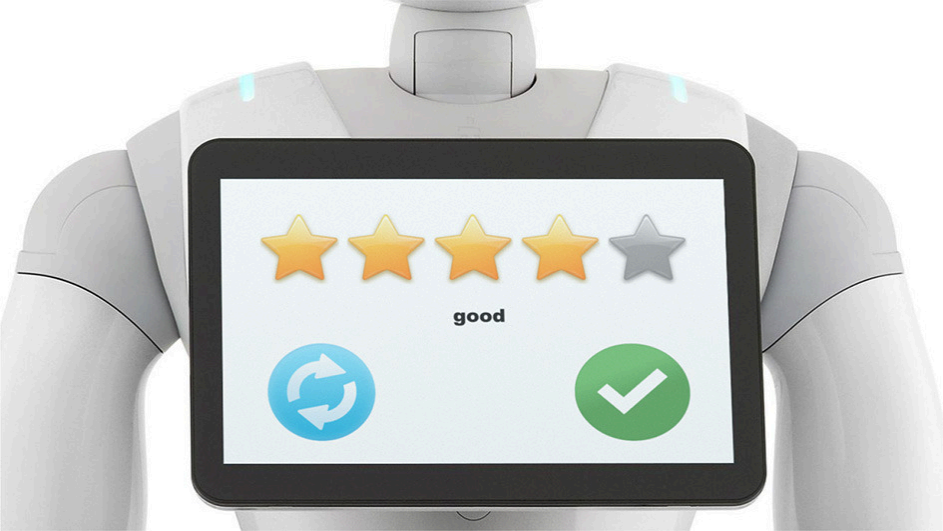
The rating GUI displayed on the robot's tablet, showing a common 5-point Likert-scale, a button to accept the chosen rating, and a button to repeat the last shown movement.

Each participant was first instructed (in German) by the experimenter. The instructions constitute a very important part of the described experiment because everything that is communicated to participants about the robot and how it learns might influence the participants' expectations and, in turn, their actions (i.e., ratings). Therefore, the instructions are described in full detail. It included the following information: The research conducted is about robot learning. The current study tests the learning of the robot Pepper and if humans are able to teach it a task, especially a game of skill called ball in cup. The goal of the game is that Pepper gets the ball into the cup with movement. During the task, Pepper will be blindfolded. The cup is in Pepper's hand and in the home position the ball is hanging still from the cup. The participant was instructed that he/she could rate each movement via a rating GUI, which was displayed on the robot's tablet (see Figure 3). The experimenter showed and explained the GUI. The participant can enter up to 5 stars for a given roll-out (as in Figure 1). The stars correspond to the ratings of (common 5-point Likert-scales) 1: not good at all, 2: not so good, 3: average, 4: good, 5: very good. A rating is confirmed via the green check mark button on the right. Another button, the replay button on the left, permitted the participant to see a movement again, if needed. When the rating was confirmed, it was transformed into a cost as cost = 6−rating to invert the scale, and was associated to the last shown movement for the CMA-ES minimization. A ready prompt screen was then shown to allow the repositioning of the ball still in the home position. After another button touch of confirmation on this screen, the robot directly showed the next roll-out.

As stated above, the camera-setup remained the same also in this study, however, the videos were only saved and used afterwards to compute ground truth. In this study, the cameras were not part of cost computation or learning. Participants were also informed of the cameras recording the movements. We told them that we would use the recordings to later follow up on what exactly the robot did. We informed participants that each participant does a fixed number of ratings at the end of which the tablet will show that the study has ended. At this point, participants were encouraged to ask any potential questions they had and informed consent was obtained from all participants prior to the experiment.

Neither did we tell participants any internals of the learning algorithm, nor did we mention any rating scheme. We also did not perform any movement to prevent priming them about correct task performance.

Then, Pepper introduced itself with its autonomous life behavior (gestures during speech and using face detection to follow the participant with its gaze). Pepper said that it wanted to learn the game blindfoldedly but did not know yet how exactly it went. It further explained that in the following it would try multiple times and the participant had to help it by telling it how good each try was. After the experimenter had blindfolded Pepper, the robot showed the movement of the initialization (see section 2.1.4).

After rating the 82 trials (the initialization + 80 generated roll-outs + the final optimized movement), each participant filled out a questionnaire on the usability of the system, and the participant's experience when teaching Pepper. A short interview was conducted that targeted participants' teaching strategies and feedback meaning.

## 3. Experimental results

### 3.1. System performance

**Figure 4:**
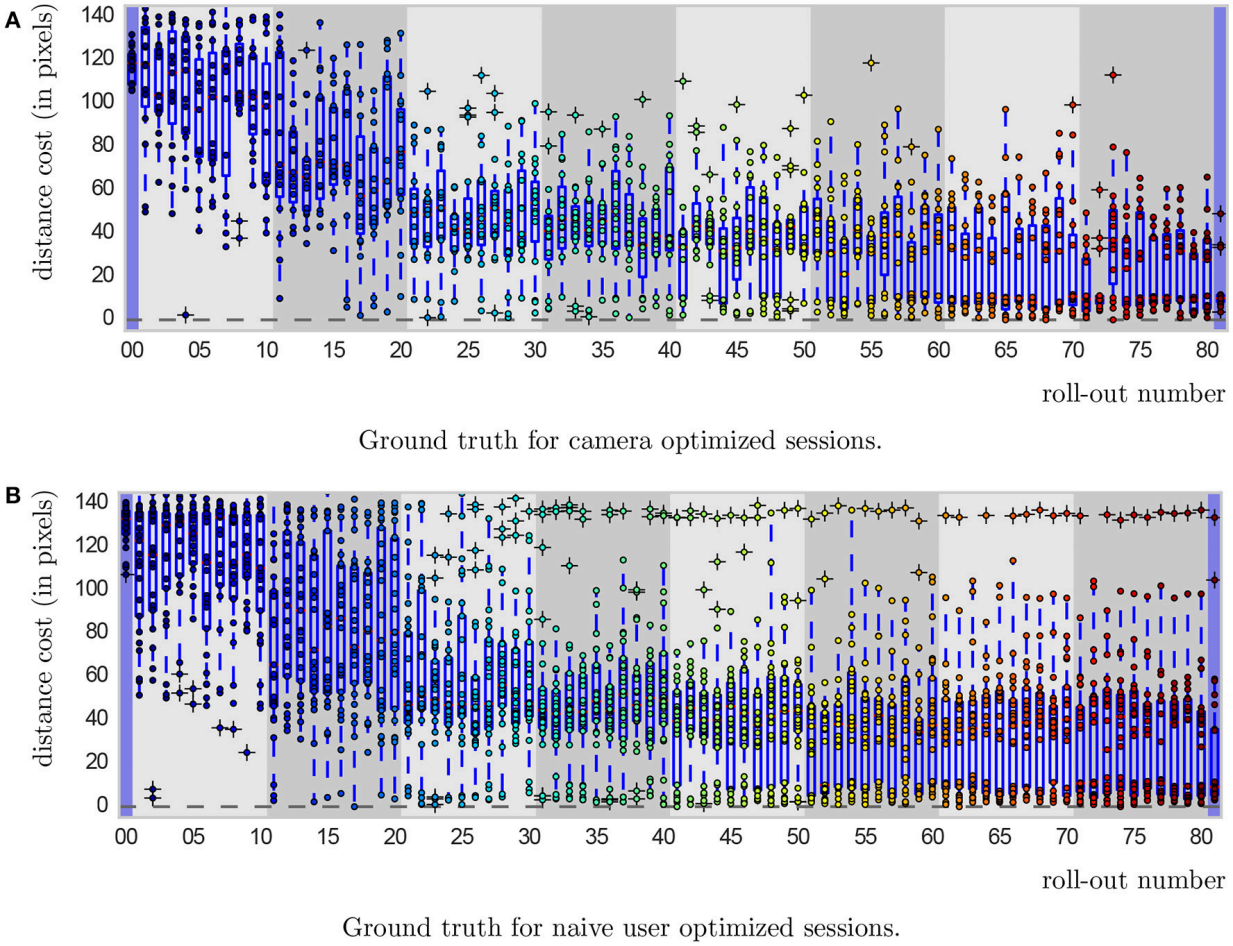
Ground truth from cameras for the 80 roll-outs in a session. First and last movements (with blue background) are initialization and final mean, respectively. Gray backgrounds indicate batches (8 in total). The central mark of box plots is the median, the lower edge of a box is the 25th percentile and the upper edge the 75th percentile, the whiskers extend to 1.5 times the interquartile range. Dots with underlying crosses lye outside the whiskers and could be considered as outliers. Successful movement executions can clearly be distinguished from unsuccessful ones, as they lie in a “band” of distance costs between 0 and around 15, corresponding to the ball lying inside the cup. The ball passing directly next to the cup resulted in a computed cost larger than 20, resulting in the clear separation that can be seen.

The system performance in the two studies is shown in Figure 4. To compare the system performance across the studies, we defined five different measures of success on the objective cost only:

- Is the final mean a hit or a miss? (Final.hit)
- The distance of the final mean in pixels (Final.dist)
- The mean distance of all roll-outs in the final batch in pixels (Batch.dist)
- The total number of hits (#hits)
- The number of roll-outs until the first hit (First.hit)

Based on these success measures, we perform statistical tests with the aim to determine what is more successful in optimizing this task, the camera setup or the naive users.

The tests did not reveal any significant differences in performance between the two. Descriptive statistics can be found in Table 2. We conducted a CHI-square test for the binary hit or miss variable of the final roll-out (Final.hit) which did not yield significant results, $\chi_{\left(1,41\right)}^{2}=1.5,p=0.221$. We conducted four independent samples t-tests for the rest of the measures. For the distance of the final mean (Final.dist), results are not significant, *t*_(35.66)_ = −1.527, *p* = 0.136. For the mean distance in roll-outs of the final batch (Batch.dist), results are not significant, *t*_(39)_ = −0.594, *p* = 0.556. For the total number of hits (#hits), results are not significant, *t*_(39)_ = 0.66, *p* = 0.513. For the number of roll-outs until the first hit (First.hit), the analysis was not significant either, *t*_((31)_ = −0.212, *p* = 0.834.

**Table 2:** Descriptive Statistics.

**Measure**	**Cam**		**HRI**	
**Final.hit**	**80%**	**hits**	**61.5%**	**hits**
	*M*	*SD*	*M*	*SD*
Final.dist (pixels)	14.39	11.21	21.89	20.15
Batch.dist (pixels)	25.88	16.00	27.82	21.66
#hits	20.27	11.84	17.96	14.97
First.hit (rollout number)	27.15	17.01	28.55	19.41

**Figure 5:**
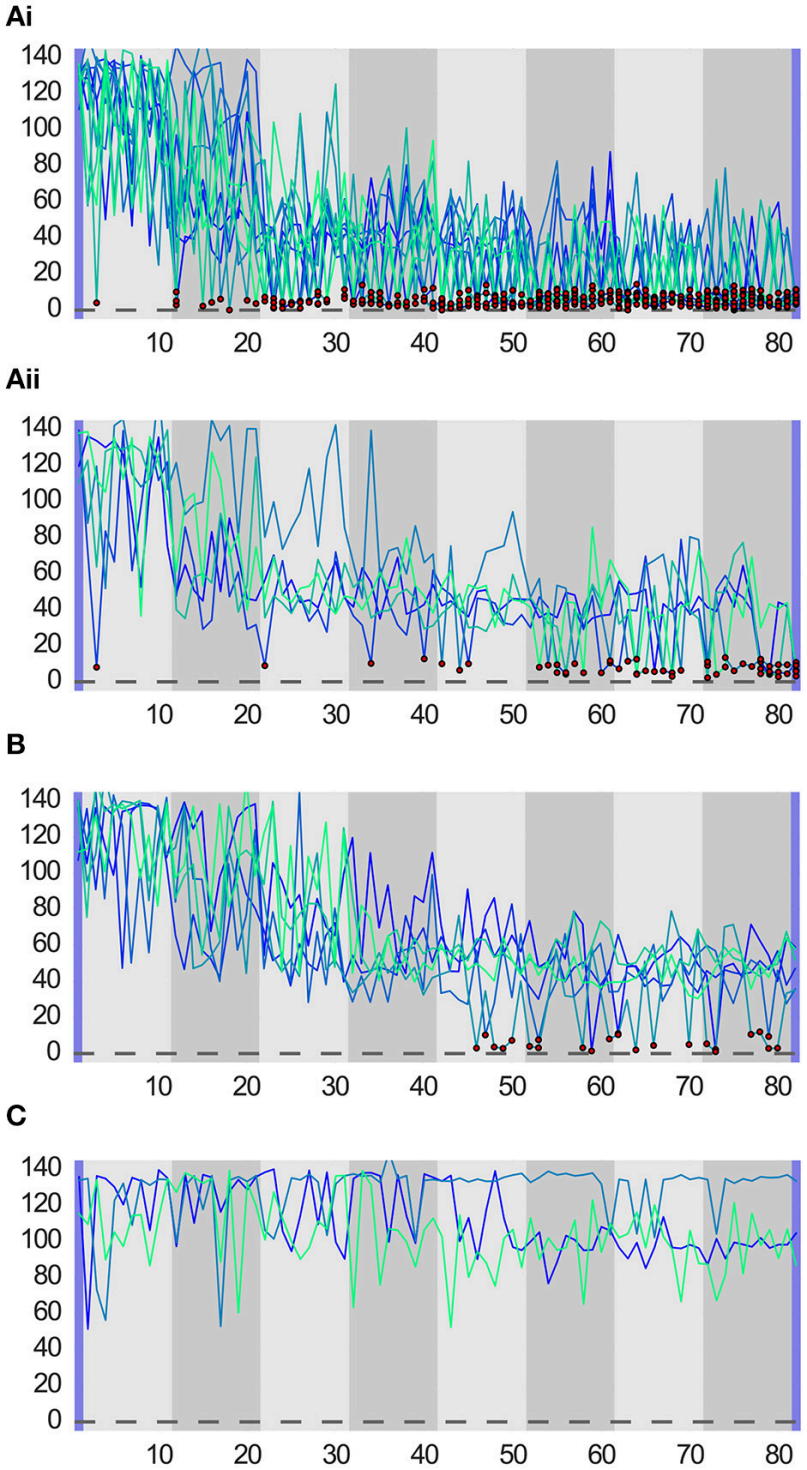
System performance for all sessions in a success category. Each line corresponds to camera obtained ground truth (i.e., automatically detected ball to cup distance in pixels) for one session (80 rollouts). Dots mark hits. Each plot corresponds to one success category: **(A.i)** successful early convergence; **(A.ii)** successful late convergence; **(B)** premature convergence; and **(C)** unsuccessful convergence.

When looking at the HRI study only, we identify three main cases of learning performance: (a) successful convergence, with sub-cases (a.i) early convergence, *N* = 12 and (a.ii) late convergence, *N* = 5; (b) premature convergence, *N* = 6; and (c) unsuccessful convergence, *N* = 3 (see Figure 5). Also in the camera optimized sessions, two out of 15 sessions showed unsuccessful convergence, which hints at important difficulties in both setups.

### 3.2. User teaching behavior

To investigate the teaching behavior of the non-expert users, we are particularly interested in the strategies that are successful or unsuccessful for learning.

#### 3.2.1. Questionnaire and interview

We first report the questionnaire and interview answers relating to the strategies of the participants in our study. This will give us a general idea about their (self-reported) teaching behavior before we analyze the actual scores. The strategies participants report in questionnaires and interviews can be categorized into five approaches.

##### 3.2.1.1. Distance from ball to cup

The majority of participants (*N* = 15) reported to use scores to rate the distance from the ball to the cup. Interestingly, all of these participants are part of sessions we identified as (a) successful convergence. This suggests that this strategy leads to success.

##### 3.2.1.2. Momentum

A few participants (*N* = 2) reported to rate the momentum of a movement. Of course at the beginning of the sessions, the momentum correlates with the distance of the ball and cup. A movement with less momentum moves the ball closer to the cup. One of the participants who reported this strategy successfully trained the robot, for the other participant, the exploration converged prematurely.

##### 3.2.1.3. Comparative ratings

A few others (*N* = 4) reported to give ratings comparing each movement to the previous one: if the movement was better than before, the rating was better and vice versa. Interestingly, sessions of participants with this teaching strategy all fall into the premature convergence category (b) described in section 3.1.

##### 3.2.1.4. Spontaneous ratings

Two participants claimed to rate the movements spontaneously, without any clear strategy (*N* = 2). For one of the two participants, exploration converged late, but successfully (a) and for the other the session was unsuccessful (c).

##### 3.2.1.5. Individual strategies

The remaining participants reported individual strategies (*N* = 3). For instance one participant in this category gave always the same score (one star) with the intention to let the robot know that it should try something completely different in order to change the movement completely. The other two strategies were not reported clearly. However, the described strategy as well as another in this category, were not successful (c). One of the participants used a strategy that lead to premature convergence (b).

#### 3.2.2. Correlation with ground truth

Based on the self-reported user strategies, we expect the successful sessions to also reflect the ‘Distance from ball to cup’ strategy in the actual scores participants gave. We test this by calculating the correlation between the participant scores and the ground truth of the robot movements. In the HRI case in general, participants received an average correlation coefficient of *M* = 0.72, *SD* = 0.20. The strategy to rate according to the distance between the ball and the cup should yield a high correlation value and thus we expect successful sessions to obtain a higher correlation coefficient than sessions with premature convergence, which in turn receives a higher correlation coefficient than unsuccessful convergence (i.e., success category *a* > *b* > *c*). Because of small sample sizes, we conduct a Kruskal-Wallis H test. There was a statistically significant difference in correlation coefficients between the three different success categories, $\chi_{\left(2\right)}^{2}=8.751,p=0.013<0.05$. An inspection of the mean ranks for the groups suggest that the successful sessions (a) had the highest correlation (*mean rank* = 16.24, *M* = 0.75, *SD* = 0.20), with the unsuccessful group (c) the lowest (*mean rank* = 2.67, *M* = 0.58, *SD* = 0.29), and prematurely converged sessions in between (*mean rank* = 11.17, *M* = 0.045, *SD* = 0.25). Pairwise *post-hoc* comparisons show a significant difference between the successful (a) and unsuccessful (c) sessions only (*p* = 0.014 < 0.05, significance value adjusted by Bonferroni correction for multiple tests). Thus the results confirm our hypothesis.

#### 3.2.3. Score data

**Figure 6:**
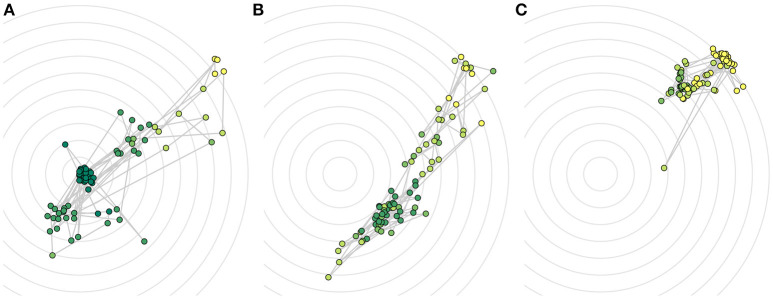
Individual visualizations for all roll-outs in one prototypical session for **(A)** successful, **(B)** premature, and **(C)** unsuccessful convergence. Colors show score given (darker shades correspond to higher scores, brighter shades correspond to lower scores). Concentric circles show equidistant positions around the cup, which is located in the center.

Prototypical plots for the three success strategies are shown in Figure 6. They corroborate and illustrate the teaching strategies we found.

Looking at individual plots of scores, we can draw a number of additional qualitative observations:

##### 3.2.3.1. Hits receive always 5 stars

We observe that a hit (i.e., the ball lands in the cup) for all participants always receives a rating of 5 stars. Though some participants reserve the 5 star rating for hits only, in general, also misses could receive a rating of 5.

##### 3.2.3.2. Rating on a global scale

One strategy we observe is to give ratings on a global scale, resulting in scores similar to the ground truth, but discrete.

##### 3.2.3.3. Rating on a local scale

Some people that rate according to the distance between ball and cup, take advantage of the full range of possible scores during the whole session and adjust their ratings according to the performance.

##### 3.2.3.4. Giving the same score multiple times

Some participants gave the same score multiple times in one batch. This could be due to perceptual difficulties. Participants often complained during the study that all movements look the same. Also this behavior could be part of a specific strategy, for example a behavior emphasizing the incorrect nature of the current kind of movement in order to get the robot to change the behavior completely (increase exploration magnitude) or a strategy that focuses on something else than the distance.

## 4. Discussion

The results of this work can be summarized with two main findings.

1. CMA-ES optimization with DMP representation works well with un-experienced, naive users, who are giving discrete feedback.
2. The main strategy users naturally apply, namely to rate according to the distance between the ball and the cup, is most successful. Relational feedback users provide, which depicts a binary relation of preference in a pair of consecutive trials, in this setup leads to premature convergence.

DMPs are an established method for open-loop state-less optimization of robot skills and have been utilized for robot learning of diverse tasks, such as for (constrained) reaching tasks (Guenter et al., 2007; Kormushev et al., 2010; Ude et al., 2010), the ball-in-the-cup game (Kober and Peters, 2009b), pick-and-place and pouring tasks (Pastor et al., 2009; Tamosiunaite et al., 2011), pancake flipping (Kormushev et al., 2010), planar biped walking (Schaal et al., 2003; Nakanishi et al., 2004), tennis swings to a fixed end-point (Ijspeert et al., 2002), T-ball batting or hitting a ball with a table tennis racket (Peters and Schaal, 2006; Calinon et al., 2010; Kober et al., 2011), pool strokes (Pastor et al., 2011), feeding a doll (Calinon et al., 2010), bi-manual manipulation of objects using chopsticks (Pastor et al., 2011), dart throwing (Kober et al., 2011), Tetherball (Daniel et al., 2012), and one-armed drumming (Ude et al., 2010).

While we so far only tested the learning in one task (the ball-in-the-cup game), our results suggest that optimization in all of these tasks, which usually entails the difficult design of cost function and sensory system, could be achieved with a simple, generic user interface even in home settings by non-expert users. Through their task knowledge, users are able to impart the goal of the task, which is not implicitly pre-programmed into the robot beforehand, without explicitly formulating or representing a cost function. Further studies involving other tasks will be needed to fully confirm this.

The discrete feedback users provide, seems to work as well as the camera setup. Even without modifications, the system is able to solve the task which could attest to (a) the robustness of this simple base-line system toward unreliable human feedback and (b) the ability of humans to adapt to the specifics of an unfamiliar learning system.

We would like to point out that the camera setup was only able to achieve the reported learning performance because of (a) the hardware used (i.e., cameras with a specific frame rate) and (b) because of the careful implementation of the cost function. As such, *naive* human teaching was not tested against a *naive* reward function but a highly tuned one. As outlined in section 2.2, the design of a suitable cost function is rarely straight-forward, and in practice requires significant adjustments to achieve the necessary precision. We believe that with a few instructions to users, system performance in this case can even be improved, and failed sessions can be prevented. We could imagine the naive users to perform even better than a cost function in some cases. For instance, toward the end of the optimization, the ball frequently hits the rim of the cup, especially, when a smaller cup is used. Because the ball moves very fast, this event is difficult to track for a vision system even with a high frame rate as it often occurs between frames. Crucially, when the ball bounces off the rim, it often travels far away from the cup and is thus assigned a high cost value by the hand-coded cost function. In contrast, humans can easily perceive this particular event, especially because it is marked with a characteristic sound, and tend to rate it with a high score. Also if the robot performs similarly bad roll-outs for some time with the ball always at a similar distance from the cup and then for the next roll-out, the ball lands at the same distance, but on the other side of the cup, the user might give a high rating to indicate the correct direction, whereas the camera setup will measure the same distance.

### 4.1. Usability of/difficulties with the current system

The optimal teaching strategy is not known for the system in this task, but it seems that most naive users are able to successfully train the robot. However, we have observed some difficulties users had with the current system.

The DMP representation does not seem to be necessarily intuitive for humans. During the optimization, it appears more difficult to get out of some regions of the parameter space than others. This is not apparent in the action space. Additionally, nine participants reported to have first given scores spontaneously and later developed a strategy, hinting at difficulties at the beginning of the sessions, because they did not have any idea how to judge the first movements as they did not know how much worse the movements could get and they did not know the magnitude of differences between movements. Apart from these initial difficulties, four participants reported to be inconsistent in their ratings at the beginning or to have started out with a rating too high. This means that there is a phase of familiarization with the system and enhanced performance can be expected for repeated teaching.

Due to the nature of CMA-ES and the way new samples are drawn from a normal distribution in the parameter space, robot performances from one batch did not differ wildly but appeared rather similar. This was confusing to some participants, as they were expecting the robot to try out a range of different movements to achieve the task. In contrast, the CMA-ES optimization resulted in rather subtle changes to the movement. As a result, some participants rated all movements from one batch with exactly the same score. This is of course critical for the CMA-ES optimization, as it gives absolutely no information about the gradient direction. This issue could also be mitigated through repeated teaching interactions and familiarity with the system.

Furthermore, with the use of CMA-ES, there is no direct impact of the ratings. Participants expected the ratings to have a direct effect on the subsequent roll-out. This lead to an exploration behavior with some participants who tested the effect of a specific rating or a specific sequence of ratings on the following roll-out. The participants reacted with surprise to the fact that after a hit, the robot again performed unsuccessful movements. The mean of the distribution in the parameter space could actually be moved directly to a hit movement, if the user had the possibility to communicate this.

The cases of premature convergence could also be prevented by, instead of CMA-ES, using an optimization algorithm with adaptive exploration, like $\text{P}{\text{I}}_{\text{CMA}}^{2}$ (Stulp and Oudeyer, 2012). Furthermore, participants were in general content with the possibility to provide feedback to the robot using a discrete scale. However, several participants commented that they would have preferred to also be able to provide verbal feedback of some form (“try with more momentum,” “try more to the left”). This supports findings by Thomaz et al. (2006) that human teachers would like to provide “guidance” signals to the learner that, in contrast to only giving feedback on the previous action, give instructions for the subsequent action. How to incorporate such feedback in the learning is subject of future work.

### 4.2. Outlook

We considered a learning algorithm without any modification or adaptation toward the human. In the following, we suggest future alterations to the system that we hypothesize to be beneficial for either system performance or usability and which can be measured systematically against the base-line.

- Giving users more instructions including information about batches in learning. We have begun to study expert teaching of this task which even outperforms camera-optimization.
- Include a button for ending optimization with the first hit. The mean is set to the current roll-out and exploration is terminated.
- Choosing an optimization algorithm with adaptive covariance estimation, to mitigate premature convergence.
- Allowing users to do the optimization twice or perform a test-run in order to alleviate skewed ratings due to wrong user expectations toward the robot.
- Studying the effect of preference-based learning on system performance and usability.

## Ethics statement

This study was carried out in accordance with the recommendations of Ethics Committee, Bielefeld University. All subjects gave written informed consent in accordance with the declaration of Helsinki. The protocol was approved by the Ethics Committee, Bielefeld University.

## Author contributions

AV and NH contributed equally to this work.

### Conflict of interest statement

The authors declare that the research was conducted in the absence of any commercial or financial relationships that could be construed as a potential conflict of interest.

## Footnotes

^1^https://youtu.be/jkaRO8J_1XI

## Supplementary material

Supplementary Video 1 Summary of the User Study on Robot Skill Learning Without a Cost Function. This video explains the idea behind the study and illustrates the progress of learning in interaction with one user in 80 rated trials.
